# Total small vessel disease score and risk of recurrent stroke: Validation in 2 large cohorts

**DOI:** 10.1212/WNL.0000000000004042

**Published:** 2017-06-13

**Authors:** Kui Kai Lau, Linxin Li, Ursula Schulz, Michela Simoni, Koon Ho Chan, Shu Leong Ho, Raymond Tak Fai Cheung, Wilhelm Küker, Henry Ka Fung Mak, Peter M. Rothwell

**Affiliations:** From the Centre for Prevention of Stroke and Dementia (K.K.L., L.L., U.S., M.S., W.K., P.M.R.), Nuffield Department of Clinical Neurosciences, University of Oxford, UK; and Division of Neurology, Department of Medicine (K.K.L., K.H.C., S.L.H., R.T.F.C.), and Department of Diagnostic Radiology (H.K.F.M.), Li Ka Shing Faculty of Medicine, University of Hong Kong.

## Abstract

**Objective:**

In patients with TIA and ischemic stroke, we validated the total small vessel disease (SVD) score by determining its prognostic value for recurrent stroke.

**Methods:**

Two independent prospective studies were conducted, one comprising predominantly Caucasian patients with TIA/ischemic stroke (Oxford Vascular Study [OXVASC]) and one predominantly Chinese patients with ischemic stroke (University of Hong Kong [HKU]). Cerebral MRI was performed and assessed for lacunes, microbleeds, white matter hyperintensities (WMH), and perivascular spaces (PVS). Predictive value of total SVD score for risk of recurrent stroke was determined and potential refinements considered.

**Results:**

In 2,002 patients with TIA/ischemic stroke (OXVASC n = 1,028, HKU n = 974, 6,924 patient-years follow-up), a higher score was associated with an increased risk of recurrent ischemic stroke (adjusted hazard ratio [HR] per unit increase: 1.32, 1.16–1.51, *p* < 0.0001; *c* statistic 0.61, 0.56–0.65, *p* < 0.0001) and intracerebral hemorrhage (ICH) (HR 1.54, 1.11–2.13, *p* = 0.009; *c* statistic 0.65, 0.54–0.76, *p* = 0.006). A higher score predicted recurrent stroke in SVD and non-SVD TIA/ischemic stroke subtypes (*c* statistic 0.67, 0.59–0.74, *p* < 0.0001 and 0.60, 0.55–0.65, *p* < 0.0001). Including burden of microbleeds and WMH and adjusting the cutoff of basal ganglia PVS potentially improved predictive power for ICH (*c* statistic 0.71, 0.60–0.81, *p*_het_ = 0.45), but not for recurrent ischemic stroke (*c* statistic 0.60, 0.56–0.65, *p*_het_ = 0.76) on internal validation.

**Conclusions:**

The total SVD score has predictive value for recurrent stroke after TIA/ischemic stroke. Prediction of recurrence in patients with nonlacunar events highlights the potential role of SVD in wider stroke etiology.

Cerebral small vessel disease (SVD) is a common cause of stroke, cognitive impairment, and gait disturbances.^1,2^ Recently, a total SVD score was proposed,^3–5^ which incorporates 4 established neuroimaging biomarkers of SVD and aims to capture the overall burden of cerebral SVD. In this score, one point is allocated to each of the following: (1) presence of lacunes, (2) presence of microbleeds, (3) moderate to severe (>10) basal ganglia (BG) perivascular spaces (PVS), and (4) severe periventricular or moderate to severe deep white matter hyperintensity (WMH).^3^ The score has been associated with age, male sex, hypertension, smoking, and lacunar stroke subtype in ischemic stroke patients.^3^ In patients with lacunar infarct, the total SVD score has also been associated with increased ambulatory blood pressure^4^ and cognitive impairment.^5^

Although the total SVD score has subsequently been shown to be associated with cognitive impairment,^6,7^ its long-term prognostic implications for recurrent stroke in patients with TIA/ischemic stroke have yet to be determined.^3^ Whether the score also predicts risk of recurrent stroke in nonlacunar stroke subtypes is unknown. Moreover, whether refinements to the score (e.g., by incorporating different weightings based on microbleed^8^ and WMH burden) may improve its predictive value has not been explored.^3,9^ Therefore, in 2 large prospective studies, 1 comprising predominantly Caucasian patients with TIA/ischemic stroke and 1 predominantly Chinese patients with ischemic stroke, we (1) validated the current total SVD score by determining its long-term prognostic implications and (2) determined whether minor refinements to the score might improve its prognostic value.

## METHODS

We prospectively studied patients with TIA/ischemic stroke from 2 cohorts: the Oxford Vascular Study (OXVASC) and the University of Hong Kong (HKU). In brief, OXVASC is an ongoing population-based study of all acute vascular events occurring within a population of 92,728 individuals, irrespective of age, who are registered with 100 general practitioners in 9 general practices of Oxfordshire, UK.^10^ This analysis includes 1,080 consecutive cases of TIA/ischemic stroke recruited from November 1, 2004, to September 30, 2014, who had a cerebral MRI. The imaging protocol of OXVASC has been described in detail elsewhere.^11,12^ Briefly, from April 1, 2002, to March 31, 2010 (phase 1), MRI and magnetic resonance angiography (MRA) was performed in selected patients when clinically indicated. From April 1, 2010 onwards (phase 2), brain MRI and MRA became the first-line imaging methods.^11^ A total of 1,076 consecutive patients who were predominantly Chinese with a diagnosis of acute ischemic stroke and received a brain MRI with MRA at the HKU MRI Unit were recruited during the period March 1, 2008, to September 30, 2014.

We collected demographic data, atherosclerotic risk factors, and details of hospitalization of index event during face-to-face interview and cross-referenced these with primary care records and hospital records in both cohorts. Cause of TIA/ischemic stroke was classified according to the modified Trial of Org 10172 in Acute Stroke Treatment (TOAST) criteria.^13^

TIA/ischemic stroke patients recruited from OXVASC were scanned predominantly (881/1,080) with 2 scanners: Achieva (Philips Healthcare, Best, the Netherlands) (493/630 patients who received a 1.5T MRI) and Magnetom Verio (Siemens health care, Munich, Germany) (388/450 patients who received a 3T MRI).^12^ All patients from HKU were scanned using a single 3T MRI scanner. Details of scan parameters are documented in table e-1 at Neurology.org. PVS were defined as small (<3 mm) punctate (if perpendicular to the plane of scan) or linear (if longitudinal to the plane of scan) hyperintensities on T2 images in the BG or centrum semiovale (CS) based on a previously validated scale.^14^ Burden of PVS was then stratified into 3 groups: <11, 11–20, and >20. The severity of white matter disease was determined for each patient according to the Fazekas scale.^15^ Cerebral microbleeds were defined as rounded, hypodense foci up to 10 mm and were differentiated from microbleed mimics based on current guidelines.^16^ The location and number of microbleeds were scored according to the Microbleed Anatomical Rating Scale.^17^ Lacunes were defined as rounded or ovoid lesions, >3 and <20 mm in diameter, in the BG, internal capsule, CS, or brainstem, with CSF signal density on T2 and fluid-attenuated inversion recovery and no increased signal on diffusion-weighted imaging.^18^

Two neuroradiologists (W.K. and H.K.F.M.) provided ongoing supervision of interpretation of the MRI throughout the study period. Definitions of neuroimaging biomarkers were based on STRIVE.^18^ The intrarater κ for 50 randomly selected scans in each center was as follows: lacunes 0.85 (OXVASC), 0.82 (HKU); microbleed burden (0, 1, 2–4, ≥5) 0.88 (OXVASC), 0.81 (HKU); periventricular WMH burden (Fazekas grade 0, 1, 2, 3) 0.66 (OXVASC), 0.69 (HKU); subcortical WMH burden (Fazekas grade 0, 1, 2, 3) 0.75 (OXVASC), 0.71 (HKU); PVS burden (<11, 11–20, >20) 0.86 (BG), 0.84 (CS) in OXVASC and 0.86 (BG), 0.72 (CS) in HKU.

All patients in OXVASC were followed up regularly by a research nurse or physician at 1, 3, 6, 12, 24, 60, and 120 months after the index event. Patients recruited from HKU were followed up by a clinician every 3–6 months, or more frequently if clinically indicated. All patients were assessed for the development of recurrent ischemic stroke or ICH. Recurrent stroke was defined as a sudden new neurologic deficit fitting the definition of ischemic stroke or ICH, occurring after a period of unequivocal neurologic stability and not attributable to cerebral edema, mass effect, or hemorrhagic transformation of the incident cerebral infarction. Patients with suspected recurrent stroke received repeat neuroimaging in the form of cranial CT or MRI to support the diagnosis. Where needed, details of clinical outcomes were supplemented by electronic or paper medical records from primary care practices, hospitals, as well as the Deaths General Register Office. The modified Rankin Scale (mRS) score of recurrent strokes was determined at 1 month after recurrent event and a disabling stroke was defined as mRS ≥3.

### Standard protocol approvals, registrations, and patient consents.

Patients gave written informed consent after an event or assent was obtained from relatives for patients who were unable to provide consent. Both cohorts were approved by the local research ethics committee.

### Statistical analysis.

We compared differences in baseline and imaging characteristics in OXVASC and HKU using Student *t* test for continuous variables and χ^2^ test for categorical variables. We determined, by Cox regression analysis, the unadjusted and adjusted (for age, sex, and vascular risk factors) risks of recurrent ischemic stroke and ICH in patients with (1) lacunes, (2) increasing burden (1, 2–4, and ≥5 vs 0) of microbleeds, (3) increasing burden (11–20 and >20 vs <11) of BG and CS PVS, and (4) increasing burden (Fazekas grade 1, 2, and 3 vs 0) of periventricular and subcortical WMH. We calculated the total SVD score for all patients^3,4^ and determined the unadjusted and adjusted (for age, sex, and vascular risk factors) odds of TIA/ischemic stroke due to SVD and subsequent risks of recurrent ischemic stroke and ICH with increasing total SVD score. The *c* statistic for area under the receiver operating characteristic curve for prediction of a recurrent stroke based on the total SVD score was calculated. Prediction of a nondisabling and disabling or fatal recurrent stroke based on increasing total SVD score was also determined.

All analyses were done with SPSS version 20 (SPSS Inc., Chicago, IL).

### Role of the funding source.

The funding source had no role in study design, data collection, data analysis, data interpretation, or writing of the report. The corresponding author had full access to all the data in the study and had the final responsibility for the decision to submit for publication.

## RESULTS

The 2 study populations contributed a total of 2,156 patients. After excluding 154 patients with incomplete clinical or imaging data, 2,002 patients (OXVASC n = 1,028, 542 TIA, 486 ischemic stroke; HKU n = 974, all ischemic stroke) were included in the final analysis. Baseline clinical and imaging characteristics of patients are shown in table 1. HKU patients had a higher proportion of male participants (*p* = 0.001) and were more likely to have hypertension and diabetes (*p* < 0.0001), while patients from OXVASC were more likely to have hyperlipidemia or a history of smoking (*p* < 0.0001).

**Table 1:**
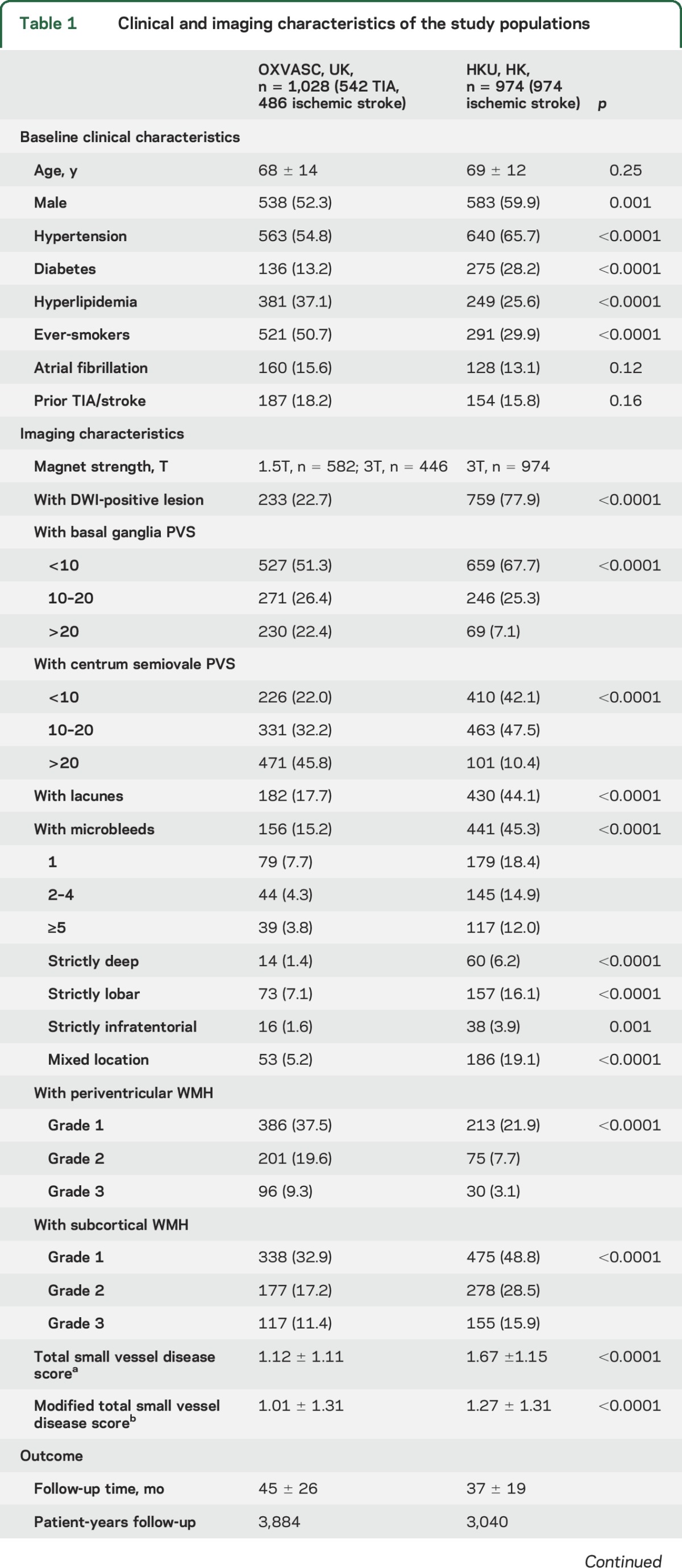

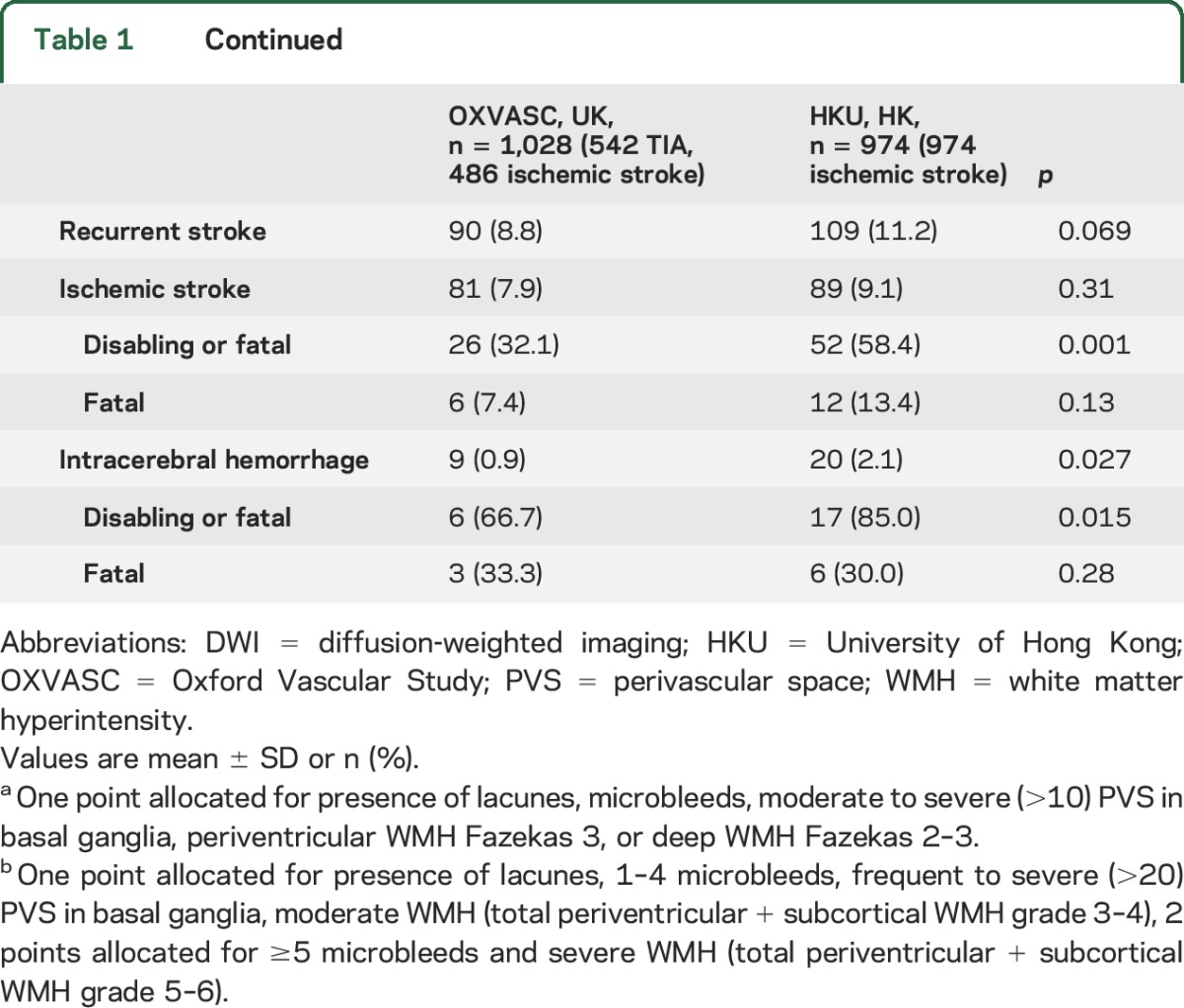
Clinical and imaging characteristics of the study populations

Patients from OXVASC had a higher burden of BG PVS, CS PVS, and periventricular WMH (*p* < 0.0001) (table 1). In contrast, those from HKU had a greater burden of lacunes, microbleeds, and subcortical WMH (*p* < 0.0001) (table 1). As expected, the total SVD score was higher in the HKU cohort than in the OXVASC cohort (*p* < 0.0001, table e-2), mainly due to a higher prevalence of microbleeds in the Asian population. These differences remained in analyses confined to OXVASC and HKU patients who received an MRI with a 3T scanner (table e-3). There were also no differences in mean total SVD scores among OXVASC patients scanned on different MRI scanners (*p* = 0.69) (table e-2).

A total of 26.8% of the study population (OXVASC n = 124/1,028, HKU n = 413/974) were classified to have TIA/ischemic stroke due to small vessel disease or occlusion by TOAST criteria (table e-4). An increasing total SVD score was associated with a greater odds of having presented at baseline with a small vessel TIA/ischemic stroke (TOAST classification) in both cohorts (OXVASC: age, sex, and vascular risk factor adjusted odds ratio per unit increase in score 2.02, 95% confidence interval 1.66–2.46, *p* < 0.0001; HKU: 1.25, 1.11–1.42, *p* = 0.0004) (table e-5).

A total of 199 recurrent strokes occurred (85.4% ischemic) after a mean follow-up of 42 ± 23 months (OXVASC: 45 ± 26 months, HKU: 37 ± 19 months, total 6,924 patient-years) (table 1). The hazard ratios (HR) of recurrent ischemic stroke and ICH based on individual markers of SVD are shown in tables e-6 through e-9 and summarized in the figure.

**Figure:**
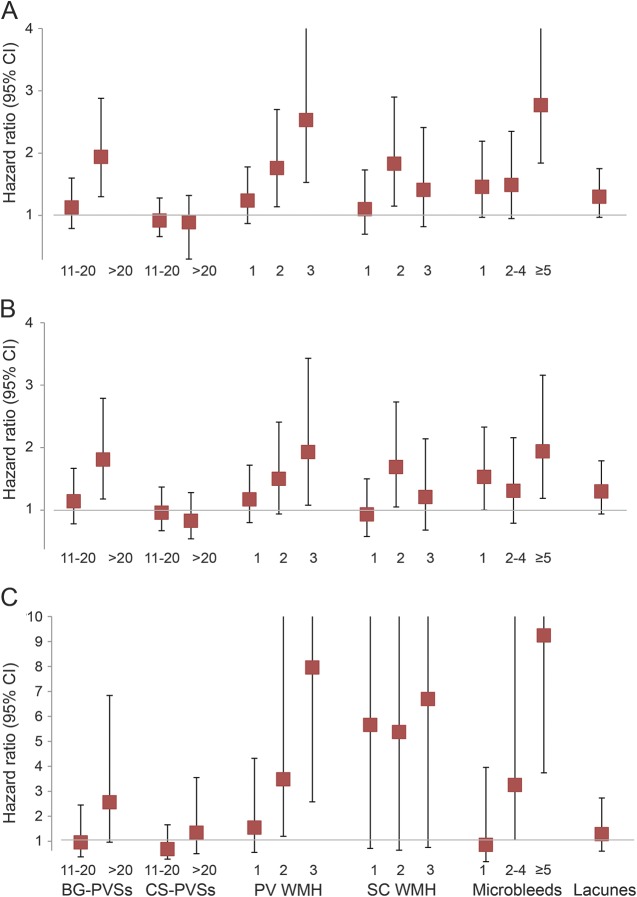
Risk of recurrent stroke, recurrent ischemic stroke, and intracerebral hemorrhage with increasing burden of small vessel disease Risk of (A) recurrent stroke, (B) recurrent ischemic stroke, and (C) intracerebral hemorrhage with increasing burden of small vessel disease. Hazard ratios adjusted for age, sex, vascular risk factors, and center and compared with patients with <11 basal ganglia (BG) perivascular spaces (PVS), <11 centrum semiovale (CS) PVS and no periventricular (PV) white matter hyperintensity (WMH), subcortical (SC) WMH, microbleeds, or lacunes, respectively. CI = confidence interval.

An increasing total SVD score was associated with an increased risk of recurrent stroke in OXVASC and HKU (OXVASC *c* statistic: 0.60, 0.54–0.67, *p* = 0.001; HKU: 0.61, 0.56–0.67, *p* = 0.0001; *p*_het_ = 0.82) (table e-10). When the 2 cohorts were pooled, patients with increasing total SVD score were at greater risk of recurrent stroke, recurrent ischemic stroke, and ICH. The multivariate adjusted HR of developing a recurrent stroke, ischemic stroke, and ICH per unit increase in score was 1.36 (1.21–1.54, *p* < 0.0001), 1.32 (1.16–1.51, *p* < 0.0001), and 1.54 (1.11–2.13, *p* = 0.009) (table 2). The *c* statistic was 0.62 (0.57–0.66, *p* < 0.0001), 0.61 (0.56–0.65, *p* < 0.0001), and 0.65 (0.54–0.76, *p* = 0.006), respectively (table 2). Patients with an increasing total SVD score were at increased risk of a recurrent nondisabling ischemic stroke (age, sex, and vascular risk factor adjusted HR per unit increase: 1.28, 1.07–1.54, *p* = 0.008) and recurrent disabling or fatal ischemic stroke (1.39, 1.14–1.69, *p* = 0.001) (table e-11). They were also at increased risk of nondisabling ICH (2.34, 1.15–4.74, *p* = 0.019). However, the risk association of total SVD score with disabling or fatal ICH did not reach statistical significance (1.42, 0.99–2.05, *p* = 0.060) (table e-11).

**Table 2:**
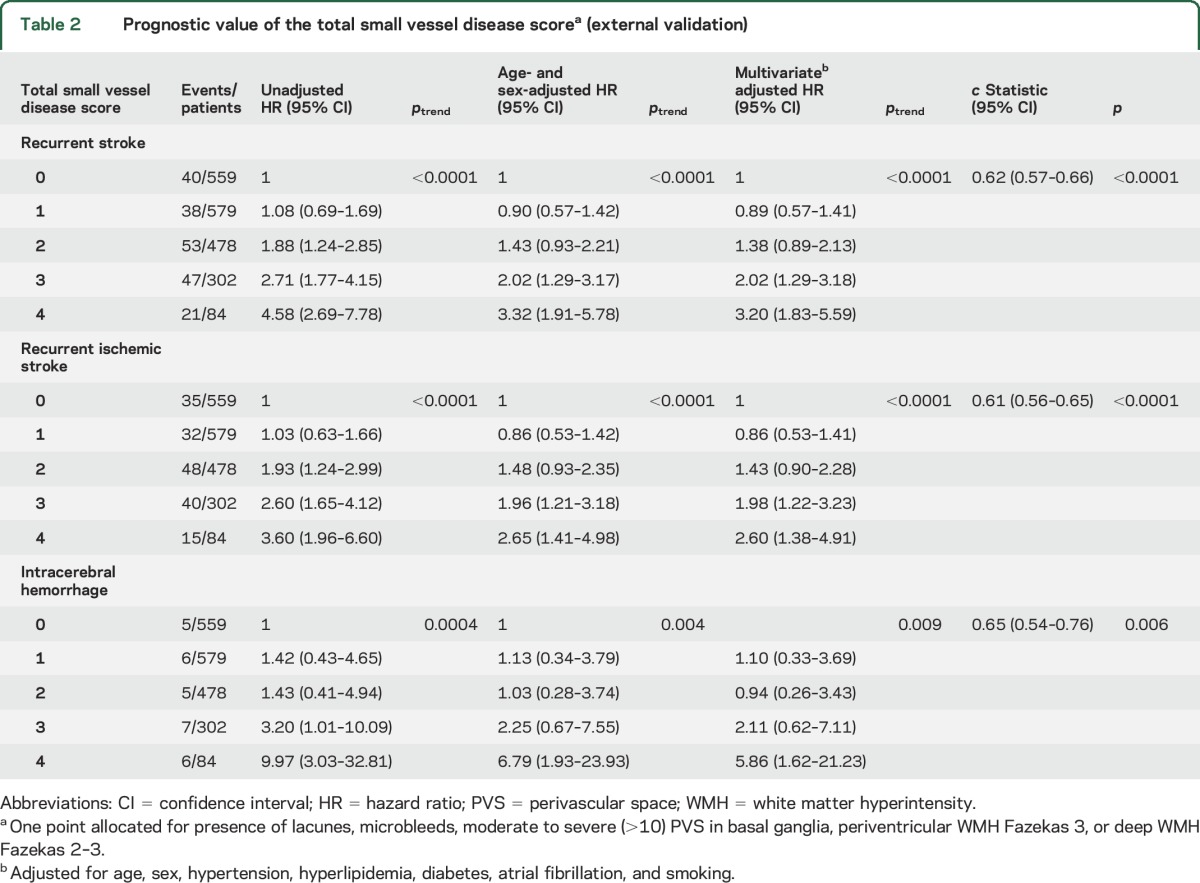
Prognostic value of the total small vessel disease score^a^ (external validation)

When all patients were stratified by their baseline TOAST subtype, the total SVD score predicted risk of recurrent stroke in both individuals presenting with a baseline TIA/ischemic stroke classified as SVD (537/2,002) (age- and sex-adjusted HR for recurrent stroke per unit increase in score in SVD subtype: 1.43 [1.12–1.83], *p* = 0.004) or non-SVD (1,465/2,002) (HR 1.39 [1.19–1.61] *p* < 0.0001 [*p*_het_ = 0.85]), the relationship of which was significant among patients with baseline TIA/ischemic stroke due to large artery atherosclerosis (large artery atherosclerosis 1.32 [1.03–1.69], cardioembolic 1.27 [0.91–1.75], undetermined 1.40 [1.00–1.95]) (table 3). Furthermore, among 1,028 OXVASC patients, the prognostic value of the total SVD score did not differ for the prediction of recurrent ischemic strokes that were classified as SVD (8/81) or non-SVD (73/81) (*p* = 0.72).

**Table 3:**
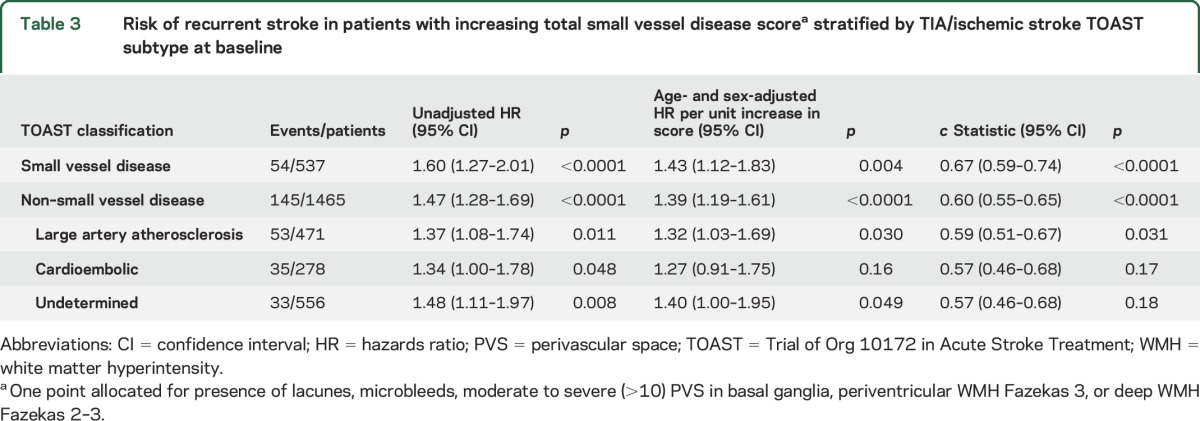
Risk of recurrent stroke in patients with increasing total small vessel disease score^a^ stratified by TIA/ischemic stroke TOAST subtype at baseline

Compared with <11 BG PVS, patients with 11–20 BG PVS were not at increased risk of recurrent stroke (adjusted HR 1.13, 0.79–1.60) (table e-9). However, risk of recurrent stroke was approximately doubled (1.94, 1.30–2.88) in patients with >20 BG PVS (table e-9 and figure). Risk of recurrent stroke was also greater with increasing burden of microbleeds (*p*_trend_ < 0.0001), especially those with ≥5 microbleeds (table e-7 and figure). Similarly, risk of recurrent stroke was also greater in patients with more severe WMH, especially those with periventricular WMH (*p*_trend_ = 0.0001) (table e-8 and figure). We therefore considered the following 3 refinements to the total SVD score: first, we allocated 1 point to those with >20 BG PVS rather than >11 BG PVS. Second, burden of microbleeds was accounted for by assigning 1 point to patients with 1–4 microbleeds and 2 points to those with ≥5 microbleeds. Third, burden of total WMH (combined periventricular and subcortical WMH) was also accounted for by allocating 1 point to those with a moderate degree of WMH (combined score of 3 or 4) and 2 points to those with severe WMH (combined score of 5 or 6).

The corresponding *c* statistic for the modified total SVD score in predicting recurrent stroke (0.62, 0.58–0.66, *p* < 0.0001) and recurrent ischemic stroke (0.60, 0.56–0.65, *p* < 0.0001) did not differ from the total SVD score in prediction of recurrent stroke (*p*_het_ = 1.00) or recurrent ischemic stroke (*p*_het_ = 0.76) (table e-12). The modified total SVD score also did not improve the prediction of nondisabling vs disabling or fatal events compared with the total SVD score (table e-11). However, the modified total SVD score might be better at identifying patients at high risk of ICH (adjusted HR per unit increase: 1.65, 1.34–2.02, *p* < 0.0001; *c* statistic 0.71, 0.60–0.81, *p* = 0.0001), but on comparison of *c* statistics, this difference was far from statistically significant (*p*_het_ = 0.45) (table e-12).

## DISCUSSION

We validated the prognostic value of the total SVD score in TIA/ischemic stroke patients in 2 large prospective cohorts involving over 2,000 Caucasian and Chinese participants with approximately 7,000 patient-years follow-up. Similar to an earlier report,^3^ the total SVD score was strongly associated with TIA/ischemic stroke due to SVD in both of these cohorts. In addition, we demonstrated that patients with a higher score were at increasing risk of a recurrent ischemic stroke and ICH and that the total SVD score predicted both nondisabling and disabling recurrent ischemic strokes. We also showed that the total SVD score predicted recurrent strokes in patients with both SVD and non-SVD subtype of TIA/ischemic stroke at baseline.

Although previous studies have demonstrated that BG PVS are markers of hypertensive angiopathy,^19,20^ the need for data on long-term prognostic implications of BG PVS has been highlighted.^18^ Our results demonstrated that compared with patients with <11 BG PVS, those with 11–20 BG PVS were not at significantly increased risk of recurrent ischemic stroke or ICH. In contrast, TIA/ischemic stroke patients with >20 BG PVS were at 1.8-fold increased risk of recurrent ischemic stroke and 2.6-fold increased risk of ICH, potentially justifying adjustment of cutoff for BG PVS in the total SVD score from >11 to >20. We also found that the risk of recurrent stroke, in particular ICH, varied substantially with burden of microbleeds, consistent with previous studies.^8^ Rather than dichotomizing the grading of microbleeds as present or absent, we considered allocating different weightings for patients with increasing burden of microbleeds. Similarly, we considered increasing the points attributed for WMH, in view of the clear stepwise increase in risk noted for increasing burden of WMH, in particular periventricular WMH. However, rather than having different cutoffs for periventricular and subcortical WMH, which may overcomplicate the score, we decided to attribute points to moderate or severe total WMH, by combining the total burden of periventricular and subcortical WMH.

Our results also support the initial decision of not incorporating CS PVS within the total SVD score. Similar to BG PVS, CS PVS have been associated with age, hypertension, WMH, and lacunes.^19–21^ However, CS PVS have also been associated with lobar microbleeds^19^ and cerebral amyloid angiopathy (CAA)^22^ and hence been hypothesized to be a neuroimaging marker of CAA by representing fluid and metabolic waste clearance dysfunction due to vascular amyloid deposition.^19,23^ While a high burden of CS PVS may be associated with subsequent risk of ICH in healthy individuals^19^ and in patients with cognitive impairment,^24^ our results demonstrate that a high burden of CS PVS was not predictive of recurrent ischemic stroke or ICH in TIA/ischemic stroke patients. Indeed, interpretation of CS PVS is often affected by the presence of concomitant subcortical WMH, which was present in 77% of patients in our cohort, and hence the usefulness of CS PVS would without doubt be limited in a TIA/ischemic stroke population.

Our study has a number of limitations. First, our 2 cohorts were different in several respects. OXVASC consists of predominantly Caucasian participants with 50% having TIA, while the HKU cohort comprises predominantly Chinese participants with ischemic stroke. Although this would probably explain some differences in stroke subtypes between the 2 populations, and there was inevitably some selection in both cohorts in relation to MRI, particularly in the earlier stages of the studies when the 2 cohorts were analyzed separately (table e-10), we were able to demonstrate that the total SVD score had very similar prognostic value in predicting risk of recurrent stroke (*c* statistic 0.60 and 0.61) with no heterogeneity. Similarly, when our results were stratified according to period of recruitment (first 5 vs latter 5 years), the total SVD score had similar prognostic value in prediction of recurrent stroke with no significant heterogeneity (*p*_het_ = 0.25). These analyses suggest that the utility of the total SVD score is robust to ethnicity and is applicable to patients with TIA and ischemic stroke. Second, our study was limited by the small number of some clinical outcomes, with only 29 ICH on long-term follow-up, among 199 total recurrent strokes. Third, the TOAST subtypes of recurrent strokes were only available among OXVASC patients. Whether the total SVD score predicts recurrent strokes of different subtypes would require further study. Fourth, patients in OXVASC were scanned on 4 different scanners over the 10-year study period. However, although this could have been a potential source of heterogeneity, total SVD scores were similar across the 4 scanners and appeared to have similar predictive value for recurrent stroke (*p*_het_ = 0.42), accepting the limited statistical power to address this for individual scanners. Overall, therefore, the robustness of our findings to different scanner types is a potential strength of the total SVD score. Fifth, any modifications to the total SVD score require further external validation in other much larger cohorts. Finally, although the total SVD score has been shown to predict cognitive impairment in the elderly population^6^ and in patients with hypertension,^7^ whether the score also predicts cognitive decline in patients with TIA/ischemic stroke remains uncertain.

## Supplementary Material

Data Supplement

## GLOSSARY

BG: basal ganglia
CAA: cerebral amyloid angiopathy
CS: centrum semiovale
HKU: University of Hong Kong
HR: hazard ratio
MRA: magnetic resonance angiography
mRS: modified Rankin Scale
OXVASC: Oxford Vascular Study
PVS: perivascular spaces
SVD: small vessel disease
TOAST: Trial of Org 10172 in Acute Stroke Treatment
WMH: white matter hyperintensity

## References

[1] Wardlaw JM, Smith C, Dichgans M. Mechanisms of sporadic cerebral small vessel disease: insights from neuroimaging. Lancet Neurol 2013;12:483–497. 23602162 10.1016/S1474-4422(13)70060-7 PMC3836247

[2] Pantoni L. Cerebral small vessel disease: from pathogenesis and clinical characteristics to therapeutic challenges. Lancet Neurol 2010;9:689–701. 20610345 10.1016/S1474-4422(10)70104-6

[3] Staals J, Makin SD, Doubal FN, Dennis MS, Wardlaw JM. Stroke subtype, vascular risk factors, and total MRI brain small-vessel disease burden. Neurology 2014;83:1228–1234. 25165388 10.1212/WNL.0000000000000837 PMC4180484

[4] Klarenbeek P, van Oostenbrugge RJ, Rouhl RP, Knottnerus IL, Staals J. Ambulatory blood pressure in patients with lacunar stroke: association with total MRI burden of cerebral small vessel disease. Stroke 2013;44:2995–2999. 23982717 10.1161/STROKEAHA.113.002545

[5] Huijts M, Duits A, van Oostenbrugge RJ, Kroon AA, de Leeuw PW, Staals J. Accumulation of MRI markers of cerebral small vessel disease is associated with decreased cognitive function: a study in first-ever lacunar stroke and hypertensive patients. Front Aging Neurosci 2013;5:72. 24223555 10.3389/fnagi.2013.00072 PMC3818574

[6] Staals J, Booth T, Morris Z, et al. Total MRI load of cerebral small vessel disease and cognitive ability in older people. Neurobiol Aging 2015;36:2806–2811. 26189091 10.1016/j.neurobiolaging.2015.06.024 PMC4706154

[7] Uiterwijk R, van Oostenbrugge RJ, Huijts M, De Leeuw PW, Kroon AA, Staals J. Total cerebral small vessel disease MRI score is associated with cognitive decline in executive function in patients with hypertension. Front Aging Neurosci 2016;8:301. 28018214 10.3389/fnagi.2016.00301 PMC5149514

[8] Wilson D, Charidimou A, Ambler G, et al. Recurrent stroke risk and cerebral microbleed burden in ischemic stroke and TIA: a meta-analysis. Neurology 2016;87:1501–1510. 27590288 10.1212/WNL.0000000000003183 PMC5075978

[9] Jickling GC, Chen C. Rating total cerebral small-vessel disease: does it add up? Neurology 2014;83:1224–1225. 25165389 10.1212/WNL.0000000000000843

[10] Rothwell PM, Coull AJ, Giles MF, et al. Change in stroke incidence, mortality, case-fatality, severity, and risk factors in Oxfordshire, UK from 1981 to 2004 (Oxford Vascular Study). Lancet 2004;363:1925–1933. 15194251 10.1016/S0140-6736(04)16405-2

[11] Li L, Yiin GS, Geraghty OC, et al. Incidence, outcome, risk factors, and long-term prognosis of cryptogenic transient ischaemic attack and ischaemic stroke: a population-based study. Lancet Neurol 2015;14:903–913. 26227434 10.1016/S1474-4422(15)00132-5 PMC5714616

[12] Simoni M, Li L, Paul NL, et al. Age- and sex-specific rates of leukoaraiosis in TIA and stroke patients: population-based study. Neurology 2012;79:1215–1222. 22955138 10.1212/WNL.0b013e31826b951e PMC3440447

[13] Adams HP Jr, Bendixen BH, Kappelle LJ, et al. Classification of subtype of acute ischemic stroke: definitions for use in a multicenter clinical trial: TOAST: Trial of Org 10172 in Acute Stroke Treatment. Stroke 1993;24:35–41. 7678184 10.1161/01.str.24.1.35

[14] Potter GM, Chappell FM, Morris Z, Wardlaw JM. Cerebral perivascular spaces visible on magnetic resonance imaging: development of a qualitative rating scale and its observer reliability. Cerebrovasc Dis 2015;39:224–231. 25823458 10.1159/000375153 PMC4386144

[15] Fazekas F, Chawluk JB, Alavi A, Hurtig HI, Zimmerman RA. MR signal abnormalities at 1.5 T in Alzheimer's dementia and normal aging. AJR Am J Roentgenol 1987;149:351–356. 3496763 10.2214/ajr.149.2.351

[16] Greenberg SM, Vernooij MW, Cordonnier C, et al. Cerebral microbleeds: a guide to detection and interpretation. Lancet Neurol 2009;8:165–174. 19161908 10.1016/S1474-4422(09)70013-4 PMC3414436

[17] Gregoire SM, Chaudhary UJ, Brown MM, et al. The Microbleed Anatomical Rating Scale (MARS): reliability of a tool to map brain microbleeds. Neurology 2009;73:1759–1766. 19933977 10.1212/WNL.0b013e3181c34a7d

[18] Wardlaw JM, Smith EE, Biessels GJ, et al. Neuroimaging standards for research into small vessel disease and its contribution to ageing and neurodegeneration. Lancet Neurol 2013;12:822–838. 23867200 10.1016/S1474-4422(13)70124-8 PMC3714437

[19] Yakushiji Y, Charidimou A, Hara M, et al. Topography and associations of perivascular spaces in healthy adults: the Kashima scan study. Neurology 2014;83:2116–2123. 25361776 10.1212/WNL.0000000000001054

[20] Zhu YC, Tzourio C, Soumare A, Mazoyer B, Dufouil C, Chabriat H. Severity of dilated Virchow-Robin spaces is associated with age, blood pressure, and MRI markers of small vessel disease: a population-based study. Stroke 2010;41:2483–2490. 20864661 10.1161/STROKEAHA.110.591586

[21] Hurford R, Charidimou A, Fox Z, Cipolotti L, Jager R, Werring DJ. MRI-visible perivascular spaces: relationship to cognition and small vessel disease MRI markers in ischaemic stroke and TIA. J Neurol Neurosurg Psychiatry 2014;85:522–525. 24249785 10.1136/jnnp-2013-305815 PMC3995332

[22] Charidimou A, Jaunmuktane Z, Baron JC, et al. White matter perivascular spaces: an MRI marker in pathology-proven cerebral amyloid angiopathy? Neurology 2014;82:57–62. 24285616 10.1212/01.wnl.0000438225.02729.04 PMC3873625

[23] Ramirez J, Berezuk C, McNeely AA, Gao F, McLaurin J, Black SE. Imaging the perivascular space as a potential biomarker of neurovascular and neurodegenerative diseases. Cell Mol Neurobiol 2016;36:289–299. 26993511 10.1007/s10571-016-0343-6 PMC11482437

[24] Martinez-Ramirez S, Pontes-Neto OM, Dumas AP, et al. Topography of dilated perivascular spaces in subjects from a memory clinic cohort. Neurology 2013;80:1551–1556. 23553482 10.1212/WNL.0b013e31828f1876 PMC3662325

