# Supplementary material for: Total small vessel disease score and risk of recurrent stroke: Validation in 2 large cohorts
Source: Neurology. 2017 Jun 13;88(24):2260–7. doi: 10.1212/WNL.0000000000004042 (PMC5567324; doi:10.1212/WNL.0000000000004042)
Supplement: Data Supplement [file supp_88_24_2260__index.html]

Total small vessel disease score and risk of recurrent stroke — Data Supplement 

# Total small vessel disease score and risk of recurrent stroke

## Data Supplement

**Neurology® data supplements are not copyedited before publication. Published editorials and translations have been copyedited.  
 © 2017 American Academy of Neurology.  
  
 Files in this Data Supplement:**

- e-Tables - Microsoft Word file
