# Supplementary material for: Total small vessel disease score and risk of recurrent stroke: Validation in 2 large cohorts
Source: Neurology. 2017 Jun 13;88(24):2260–7. doi: 10.1212/WNL.0000000000004042 (PMC5567324; doi:10.1212/WNL.0000000000004042)
Supplement: Data Supplement [file supp_WNL.0000000000004042_e-Tables.docx]

**Supplementary Tables:**

Table e-1. Imaging sequence parameters of the OXVASC and HKU cohorts

Table e-2. Distribution of Total Small Vessel Disease Score amongst OXVASC and HKU patients

Table e-3. Clinical and imaging characteristics in OXVASC and HKU patients who were scanned by a 3T MRI

Table e-4. Aetiology of TIA / ischaemic strokes according to TOAST classification in the OXVASC and HKU cohorts

Table e-5. Association of Total Small Vessel Disease Score with TOAST subtype of baseline TIA / ischaemic stroke: small vessel events vs. other subtypes

Table e-6. Cox regression analyses of risk of recurrent stroke for lacunes vs. no lacunes

Table e-7. Cox regression analyses of risk of recurrent stroke for different burden of microbleeds vs. no microbleeds

Table e-8. Cox regression analyses of risk of recurrent stroke for different burden of white matter hyperintensity vs. no white matter hyperintensity

Table e-9. Cox regression analyses of risk of recurrent stroke for different burden of perivascular spaces vs. <11 perivascular spaces

Table e-10. Prognostic value of the Total Small Vessel Disease Score in prediction of recurrent strokes in OXVASC and HKU (external validation)

Table e-11. Prognostic value of the Total Small Vessel Disease Score and modified Total Small Vessel Disease Score in prediction of non-disabling and disabling or fatal recurrent strokes

Table e-12. Prognostic value of modified Total Small Vessel Disease Score (internal validation)

| **MR parameters** | **HKU**  **Achieva, Philips Healthcare** | **OXVASC scanner 1**  **Magnetom Verio, Siemens Healthcare** | **OXVASC scanner 2**  **Discovery MR750, GE Healthcare** | **OXVASC scanner 3**  **Achieva, Philips Healthcare** | **OXVASC scanner 4**  **Signa HDxt, GE Healthcare** |
| --- | --- | --- | --- | --- | --- |
| **Patients scanned** | 1076 | 388 | 62 | 493 | 137 |
| **Field strength (T)** | 3 | 3 | 3 | 1.5 | 1.5 |
| **T1W TR/TE/TI (ms)** | 2000/20/800 | 2000/1.94/880 | - | 701/16 | - |
| **T2W TR/TE (ms)** | 2377/80 | 6000/96 | 5800/94 | 5061/100 | 3760/100 |
| **FLAIR TR/TE/TI (ms) (3D)** | 4800/282/1650 | 9000/88/2500 | 9600/130/2350 | 11000/140/2800 | 8080/112/2200 |
| **Diffusion TR/TE (ms)** | 2874/46 | 5300/91 | 6000/84 | 2891/73 | 6100/71 |
| **GRE / SWI TR/TE (ms) (3D)** | SWI 28/23 | GRE 504/15 | GRE 500/20 | GRE 694/23 | GRE 560/25 |
| **Pixel bandwidth (Hz)** | 218.5 (T1W)  350.7 (T2W)  144.7 (FLAIR)  40.2 (Diffusion)  455.7 (SWI) | 240 (T1W)  220 (T2W)  202 (FLAIR)  1374 (Diffusion)  200 (GRE) | -  50 (T2W)  41.7 (FLAIR)  250 (Diffusion)  31.3 (GRE) | 87.4 (T1W)  88.5 (T2W)  375 (FLAIR)  25.3 (Diffusion)  109.3 (GRE) | -  47.6 (T2W)  31.3 (FLAIR)  -  75 (GRE) |
| **Matrix** | 308x207 (T1W)  308x235 (T2W)  228x227 (FLAIR)  112x87 (Diffusion)  256x224 (SWI) | 256x256 (T1W)  320x320 (T2W)  192x192 (FLAIR)  130x130 (Diffusion)  320x256 (GRE) | -  512 (T2W)  384x224 (FLAIR)  128x128 (Diffusion)  288x224 (GRE) | 118x214 (T1W)  356x193 (T2W)  236x159 (FLAIR)  97x84 (Diffusion)  256x163 (GRE) | 416x256 (T2W)  256x224 (FLAIR)  128x128 (Diffusion)  288x192 (GRE) |
| **No. of slices** | 25 (T1W)  25 (T2W)  30 (FLAIR)  25 (Diffusion)  25 (SWI) | 208 (T1W)  25 (T2W)  50 (FLAIR)  25 (Diffusion)  25 (GRE) | 25 | 25 (T1W)  25 (T2W)  28 (FLAIR)  25 (Diffusion)  22 (GRE) | 25 |
| **Slice thickness (mm)** | 5 (T1W)  5 (T2W)  5 (FLAIR coronal)  2.5 (FLAIR axial)  5 (Diffusion)  5 (SWI) | 1 (T1W)  5 (T2W)  3 (FLAIR)  5 (Diffusion)  5 (GRE) | 5 | 5 | 5 |
| **Inter-slice gap (mm)** | 0.5 (T1W)  0.5(T2W)  0.5 (FLAIR coronal)  0 (FLAIR axial)  0.5 (Diffusion)  0.5 (SWI) | 0 (T1W)  1 (T2W)  0 (FLAIR coronal)  1 (Diffusion)  1 (GRE) | 1 | 1 | 1 |
| **Voxel size (mm^3^)** | 0.75x0.95x5.0 (T1W)  0.75x0.76x5.0 (T2W)  1.10x1.10x1.12 (FLAIR)  2.05x2.64x5.0 (Diffusion)  0.90x0.90x2.00 (SWI) | 1.0x1.0x1.0 (T1W)  0.8x0.8x5.0 (T2W)  1.0x1.0x3.0 (FLAIR)  1.8x1.8x5.0 (Diffusion)  0.9x0.8x5.0 (GRE) | - | 0.53x0.53x5.0 (T1W)  0.65x0.65x5.0 (T2W)  0.82x0.81x5.0 (FLAIR)  1.74x1.73x5.0 (Diffusion)  0.90x0.90x5.0 (GRE) | - |

Table e-1. Imaging sequence parameters of the OXVASC and HKU cohorts

Table e-2. Distribution of Total Small Vessel Disease Score amongst OXVASC and HKU patients

|  | **HKU**  **Achieva, Philips Healthcare (n=974)** | **OXVASC scanner 1**  **Magnetom Verio, Siemens Healthcare (n=384)** | **OXVASC scanner 2**  **Discovery MR750, GE Healthcare (n=62)** | **OXVASC scanner 3**  **Achieva, Philips Healthcare (n=489)** | **OXVASC scanner 4**  **Signa HDxt, GE Healthcare (n=93)** |
| --- | --- | --- | --- | --- | --- |
| Total Small Vessel Disease Score |  |  |  |  |  |
| 0 (%) | 172 (17.7) | 114 (29.7) | 24 (38.7) | 208 (42.5) | 41 (44.1) |
| 1 (%) | 286 (29.4) | 145 (37.8) | 13 (21.0) | 114 (23.3) | 21 (22.6) |
| 2 (%) | 263 (27.0) | 94 (24.5) | 15 (24.2) | 86 (17.6) | 20 (21.5) |
| 3 (%) | 199 (20.4) | 24 (6.3) | 7 (11.3) | 61 (12.5) | 11 (11.8) |
| 4 (%) | 54 (5.5) | 7 (1.8) | 3 (4.8) | 20 (4.1) | 0 (0.0) |

Table e-3. Clinical and imaging characteristics in OXVASC and HKU patients who were scanned by a 3T MRI

|  | **OXVASC, UK**  **n=446**  **(267 TIA , 179 ischaemic stroke)** | **HKU, HK**  **n=974**  **974 ischaemic stroke** | **p** |
| --- | --- | --- | --- |
| **Baseline clinical characteristics** |  |  |  |
| Mean age, yr (SD) | 70 (14) | 69 (12) | 0.39 |
| Males (%) | 235 (52.7) | 583 (59.9) | 0.011 |
| Hypertension (%) | 237 (53.1) | 640 (65.7) | <0.0001 |
| Diabetes (%) | 53 (11.9) | 275 (28.2) | <0.0001 |
| Hyperlipidaemia (%) | 154 (34.5) | 249 (25.6) | 0.001 |
| Ever-smokers (%) | 190 (42.6) | 291 (29.9) | <0.0001 |
| Atrial fibrillation (%) | 69 (15.5) | 128 (13.1) | 0.24 |
| Prior TIA / stroke (%) | 77 (17.3) | 154 (15.8) | 0.49 |
|  |  |  |  |
| **Imaging characteristics** |  |  |  |
| N with DWI positive lesion (%) | 101 (22.6) | 759 (77.9) | <0.0001 |
| N with basal ganglia PVSs (%) |  |  |  |
| <10 (%) | 181 (40.6) | 659 (67.7) | <0.0001 |
| 10-20 (%) | 150 (33.6) | 246 (25.3) |  |
| >20 (%) | 115 (25.8) | 69 (7.1) |  |
| N with centrum semiovale PVSs (%) |  |  |  |
| <10 (%) | 60 (13.5) | 410 (42.1) | <0.0001 |
| 10-20 (%) | 138 (30.9) | 463 (47.5) |  |
| >20 (%) | 248 (55.6) | 101 (10.4) |  |
| N with lacunes (%) | 58 (13.0) | 430 (44.1) | <0.0001 |
| N with microbleeds (%) | 64 (14.3) | 441 (45.3) | <0.0001 |
| 1 microbleed (%) | 35 (7.8) | 179 (18.4) |  |
| 2-4 microbleeds (%) | 17 (3.8) | 145 (14.9) |  |
| ≥5 microbleeds (%) | 12 (2.7) | 117 (12.0) |  |
| Periventricular WMH grade (%) |  |  |  |
| Grade 1 (%) | 182 (40.8) | 213 (21.9) | <0.0001 |
| Grade 2 (%) | 89 (20.0) | 75 (7.7) |  |
| Grade 3 (%) | 31 (7.0) | 30 (3.1) |  |
| N with subcortical WMH (%) |  |  |  |
| Grade 1 (%) | 141 (31.6) | 475 (48.8) | <0.0001 |
| Grade 2 (%) | 70 (15.7) | 278 (28.5) |  |
| Grade 3 (%) | 53 (11.9) | 155 (15.9) |  |
| Mean Total Small Vessel Disease Score^a^ | 1.14±1.01 | 1.67±1.15 | <0.0001 |
| Mean modified Total Small Vessel Disease Score^b^ | 0.94±1.24 | 1.27±1.31 | <0.0001 |

Abbreviations: TIA=transient ischaemic attack; DWI=diffusion weighted imaging; PVS=perivascular space; WMH=white matter hyperintensity
^a^1 point allocated for: presence of lacunes, microbleeds, moderate-severe (>10) PVSs in basal ganglia, periventricular WMH Fazekas 3 and/or deep WMH Fazekas 2-3
^b^1 point allocated for: presence of lacunes, 1-4 microbleeds, frequent-severe (>20) PVSs in basal ganglia, moderate WMH (total periventricular + subcortical WMH grade 3-4), 2 points allocated for ≥5 microbleeds and severe WMH (total periventricular + subcortical WMH grade 5-6)

Table e-4. Aetiology of TIA / ischaemic strokes according to TOAST classification in the OXVASC and HKU cohorts

|  | **OXVASC, UK**  **n=1028**  **(542 TIA, 486 ischaemic stroke)** | **HKU, HK**  **n=974**  **(974 ischaemic stroke)** | **All**  **n=2002** |
| --- | --- | --- | --- |
| Small vessel disease (%) | 124 (12.1) | 413 (42.4) | 537 (26.8) |
| Large artery atherosclerosis (%) | 137 (13.3) | 334 (34.3) | 471 (23.5) |
| Cardio-embolic (%) | 160 (15.6) | 118 (12.1) | 278 (13.9) |
| Undetermined (%) | 514 (50.0) | 42 (4.3) | 556 (27.8) |
| Multiple (%) | 35 (3.4) | 28 (2.9) | 63 (3.1) |
| Unknown (%) | 26 (2.5) | 22 (2.3) | 48 (2.4) |
| Others (%) | 32 (3.1) | 17 (1.7) | 49 (2.4) |

Abbreviations: TIA=transient ischaemic attack, TOAST=Trial of Org 10172 in Acute Stroke Treatment

Table e-5. Association of Total Small Vessel Disease Score^a^ with TOAST subtype of baseline TIA / ischaemic stroke: small vessel events vs. other subtypes

| **Total Small Vessel Disease Score** | **Events / patients** | **Unadjusted HR (95% CI)** | **p_trend_** | **Age and sex adjusted HR (95% CI)** | **p_trend_** | **Multi-variate^b^ adjusted HR (95% CI)** | **p_trend_** |
| --- | --- | --- | --- | --- | --- | --- | --- |
| **OXVASC** |  |  |  |  |  |  |  |
| 0 | 35/387 | 1 | <0.0001 | 1 | <0.0001 | 1 | <0.0001 |
| 1 | 27/293 | 1.02 (0.60-1.73) |  | 1.67 (0.94-2.99) |  | 1.84 (1.02-3.32) |  |
| 2 | 29/215 | 1.57 (0.93-2.65) |  | 3.11 (1.68-5.77) |  | 3.78 (1.99-7.19) |  |
| 3 | 22/103 | 2.73 (1.52-4.91) |  | 5.93 (2.97-11.84) |  | 7.07 (3.47-14.42) |  |
| 4 | 11/30 | 5.82 (2.57-13.22) |  | 13.21 (5.28-33.04) |  | 20.09 (7.45-54.20) |  |
| **HKU** |  |  |  |  |  |  |  |
| 0 | 59/172 | 1 | 0.002 | 1 | <0.0001 | 1 | 0.0004 |
| 1 | 116/286 | 1.31 (0.88-1.94) |  | 1.38 (0.93-2.05) |  | 1.21 (0.80-1.82) |  |
| 2 | 117/263 | 1.54 (1.03-2.29) |  | 1.77 (1.18-2.67) |  | 1.58 (1.04-2.42) |  |
| 3 | 90/199 | 1.58 (1.04-2.41) |  | 1.94 (1.25-3.00) |  | 1.69 (1.07-2.66) |  |
| 4 | 31/54 | 2.58 (1.38-4.82) |  | 3.35 (1.76-6.37) |  | 3.20 (1.62-6.35) |  |

^a^1 point allocated for: presence of lacunes, microbleeds, moderate-severe (>10) PVSs in basal ganglia, periventricular WMH Fazekas 3 and/or deep WMH Fazekas 2-3

^b^Adjusted for age, sex, hypertension, hyperlipidaemia, diabetes, atrial fibrillation and smoking

Abbreviations: TIA=transient ischaemic attack, TOAST=Trial of Org 10172 in Acute Stroke Treatment; HR=hazards ratio, CI=confidence interval; PVS=perivascular space; WMH=white matter hyperintensity

Table e-6. Cox regression analyses of risk of recurrent stroke for lacunes vs. no lacunes

|  | **Univariate HR (95% CI)^a^** | **p** | **HR (95% CI) adjusted for age and sex ^a^** | **p** | **HR (95% CI) adjusted for age, sex and vascular risk factors ^a,b^** | **p** |
| --- | --- | --- | --- | --- | --- | --- |
| **Recurrent stroke** |  |  |  |  |  |  |
| OXVASC | 1.62 (1.01-2.60) | 0.047 | 1.47 (0.91-2.38) | 0.12 | 1.39 (0.86-2.24) | 0.18 |
| HKU | 1.28 (0.88-1.86) | 0.20 | 1.27 (0.87-1.86) | 0.21 | 1.28 (0.87-1.87) | 0.21 |
| Combined^c^ | 1.40 (1.04-1.89) | 0.027 | 1.34 (0.99-1.80) | 0.056 | 1.30 (0.97-1.75) | 0.084 |
| **Ischaemic stroke** |  |  |  |  |  |  |
| OXVASC | 1.55 (0.93-2.57) | 0.091 | 1.44 (0.86-2.39) | 0.17 | 1.33 (0.80-2.22) | 0.28 |
| HKU | 1.32 (0.87-2.00) | 0.19 | 1.33 (0.87-2.02) | 0.18 | 1.34 (0.88-2.05) | 0.17 |
| Combined^c^ | 1.42 (1.02-1.96) | 0.036 | 1.34 (0.97-1.86) | 0.072 | 1.30 (0.94-1.79) | 0.12 |
| **Intracerebral haemorrhage** |  |  |  |  |  |  |
| OXVASC | 2.18 (0.54-8.70) | 0.27 | 1.61 (0.40-6.55) | 0.50 | 1.52 (0.35-6.73) | 0.58 |
| HKU | 1.06 (0.44-2.56) | 0.90 | 1.03 (0.42-2.49) | 0.95 | 1.04 (0.43-2.53) | 0.93 |
| Combined^c^ | 1.29 (0.60-2.76) | 0.52 | 1.26 (0.59-2.69) | 0.55 | 1.28 (0.60-2.73) | 0.53 |

^a^Compared with no lacunes as reference

^b^Hypertension, hyperlipidaemia, diabetes mellitus, atrial fibrillation smoking history

^c^Also adjusted for Centre

Abbreviations: HR=hazard ratio, CI=confidence interval

Table e-7. Cox regression analyses of risk of recurrent stroke for different burden of microbleeds vs. no microbleeds

|  | **Unadjusted HR (95% CI)^a^** | | | | **HR (95% CI) adjusted for age and sex ^a^** | | | | **HR (95% CI) adjusted for age, sex and vascular risk factors ^a,b^** | | | |
| --- | --- | --- | --- | --- | --- | --- | --- | --- | --- | --- | --- | --- |
| **Microbleeds** | **1** | **2-4** | **≥5** | **p_trend_** | **1** | **2-4** | **≥5** | **p_trend_** | **1** | **2-4** | **≥5** | **p_trend_** |
| **Recurrent stroke** |  |  |  |  |  |  |  |  |  |  |  |  |
| OXVASC | 2.08  (1.10-3.95) | 2.07  (0.90-4.78) | 3.28  (1.57-6.85) | 0.0002 | 1.84  (0.96-3.52) | 1.87  (0.80-4.35) | 2.83  (1.34-5.98) | 0.002 | 1.81  (0.95-3.47) | 1.73  (0.74-4.06) | 3.00  (1.41-6.36) | 0.002 |
| HKU | 1.42  (0.84-2.38) | 1.68  (0.98-2.87) | 2.88  (1.76-4.71) | <0.0001 | 1.32  (0.78-2.22) | 1.43  (0.83-2.46) | 2.52  (1.54-4.12) | 0.001 | 1.39  (0.82-2.35) | 1.41  (0.82-2.44) | 2.82  (1.72-4.65) | 0.0002 |
| Combined^c^ | 1.64  (1.09-2.46) | 1.84  (1.17-2.88) | 3.09  (2.06-4.64) | <0.0001 | 1.47  (0.98-2.21) | 1.58  (1.01-2.49) | 2.66  (1.77-4.00) | <0.0001 | 1.46  (0.97-2.19) | 1.49  (0.95-2.35) | 2.77  (1.84-4.16) | <0.0001 |
| **Ischaemic stroke** |  |  |  |  |  |  |  |  |  |  |  |  |
| OXVASC | 2.03  (1.04-3.97) | 1.48  (0.54-4.07) | 3.10  (1.42-6.79) | 0.002 | 1.83  (0.93-3.62) | 1.33  (0.48-3.70) | 2.77  (1.25-6.15) | 0.010 | 1.76  (0.89-3.49) | 1.16  (0.42-3.24) | 2.87  (1.29-6.39) | 0.014 |
| HKU | 1.53  (0.89-2.62) | 1.58  (0.88-2.83) | 1.76  (0.95-3.24) | 0.033 | 1.42  (0.83-2.43) | 1.34  (0.75-2.41) | 1.53  (0.83-2.82) | 0.13 | 1.51  (0.87-2.59) | 1.34  (0.74-2.43) | 1.81  (0.97-3.35) | 0.057 |
| Combined^c^ | 1.72  (1.13-2.62) | 1.64  (1.00-2.69) | 2.14  (1.32-3.49) | 0.001 | 1.54  (1.01-2.35) | 1.41  (0.85-2.32) | 1.85  (1.14-3.01) | 0.008 | 1.53  (1.00-2.33) | 1.31  (0.79-2.16) | 1.94  (1.19-3.16) | 0.007 |
| **Intracerebral haemorrhage** |  |  |  |  |  |  |  |  |  |  |  |  |
| OXVASC | 2.44  (0.28-20.91) | 8.87  (1.72-45.80) | 4.91  (0.57-42.26) | 0.034 | 1.88  (0.22-16.35) | 10.12  (1.88-54.39) | 3.45  (0.39-30.44) | 0.034 | 1.82  (0.19-17.20) | 11.42  (1.83-71.19) | 5.20  (0.52-52.34) | 0.024 |
| HKU | 0.58  (0.07-4.93) | 2.29  (0.55-9.59) | 10.85  (3.77-31.24) | <0.0001 | 0.54  (0.06-4.64) | 2.05  (0.49-8.63) | 9.84  (3.39-28.54) | <0.0001 | 0.58  (0.07-4.99) | 2.07  (0.49-8.86) | 9.18  (3.13-26.92) | <0.0001 |
| Combined^c^ | 0.98  (0.21-4.57) | 3.47  (1.14-10.61) | 10.55  (4.33-25.71) | <0.0001 | 0.88  (0.19-4.09) | 3.06  (0.99-9.43) | 8.99  (3.67-22.05) | <0.0001 | 0.85  (0.18-3.96) | 3.25  (1.05-10.06) | 9.24  (3.74-22.81) | <0.0001 |

^a^Compared with no microbleeds as reference

^b^Hypertension, hyperlipidaemia, diabetes mellitus, atrial fibrillation smoking history

^c^Also adjusted for Centre

Abbreviations: HR=hazard ratio, CI=confidence interval

Table e-8. Cox regression analyses of risk of recurrent stroke for different burden of white matter hyperintensity vs. no white matter hyperintensity

|  | **Unadjusted HR (95% CI)^a^** | | | | **HR (95% CI) adjusted for age and sex ^a^** | | | | **HR (95% CI) adjusted for age, sex and vascular risk factors^a,b^** | | | |
| --- | --- | --- | --- | --- | --- | --- | --- | --- | --- | --- | --- | --- |
| **Periventricular WMH** | **1** | **2** | **3** | **p_trend_** | **1** | **2** | **3** | **p_trend_** | **1** | **2** | **3** | **p_trend_** |
| **Recurrent stroke** |  |  |  |  |  |  |  |  |  |  |  |  |
| OXVASC | 2.42  (1.33-4.43) | 3.31  (1.76-6.32) | 4.59  (2.24-9.42) | p<0.0001 | 2.31  (1.22-4.39) | 3.09  (1.52-6.28) | 4.21  (1.90-9.36) | 0.0002 | 2.03  (1.07-3.86) | 2.79  (1.38-5.66) | 4.14  (1.88-9.10) | 0.0002 |
| HKU | 1.21  (0.76-1.92) | 1.80  (0.99-3.27) | 3.12  (1.49-6.51) | 0.002 | 0.91  (0.57-1.47) | 1.25  (0.68-2.30) | 2.43  (1.15-5.12) | 0.089 | 0.94  (0.59-1.52) | 1.51  (0.81-2.82) | 2.57  (1.21-5.44) | 0.030 |
| Combined^c^ | 1.54  (1.09-2.17) | 2.21  (1.48-3.32) | 3.30  (2.04-5.33) | <0.0001 | 1.27  (0.89-1.81) | 1.69  (1.11-2.59) | 2.39  (1.44-3.98) | 0.0004 | 1.24  (0.87-1.78) | 1.76  (1.14-2.70) | 2.53  (1.53-4.21) | 0.0001 |
| **Ischaemic stroke** |  |  |  |  |  |  |  |  |  |  |  |  |
| OXVASC | 2.27  (1.23-4.16) | 2.85  (1.47-5.53) | 3.28  (1.50-7.16) | 0.001 | 2.25  (1.17-4.31) | 2.78  (1.34-5.77) | 3.13  (1.32-7.40) | 0.005 | 1.91  (1.00-3.67) | 2.43  (1.17-5.02) | 3.00  (1.28-6.99) | 0.006 |
| HKU | 1.12  (0.67-1.88) | 1.43  (0.71-2.89) | 2.70  (1.16-6.28) | 0.038 | 0.82  (0.49-1.39) | 0.97  (0.47-2.00) | 2.03  (0.86-4.77) | 0.48 | 0.86  (0.51-1.46) | 1.28  (0.62-2.65) | 2.22  (0.94-5.25) | 0.19 |
| Combined^c^ | 1.48  (1.02-2.13) | 1.91  (1.22-2.97) | 2.57  (1.49-4.44) | <0.0001 | 1.21  (0.83-1.77) | 1.44  (0.91-2.30) | 1.83  (1.03-3.27) | 0.027 | 1.17  (0.80-1.72) | 1.50  (0.94-2.41) | 1.93  (1.08-3.43) | 0.015 |
| **Intracerebral haemorrhage** |  |  |  |  |  |  |  |  |  |  |  |  |
| OXVASC^d^ | - | - | - |  | - | - | - |  | - | - | - |  |
| HKU | 1.67  (0.56-4.99) | 3.80  (1.17-12.36) | 5.44  (1.17-25.18) | 0.006 | 1.49  (0.48-4.61) | 3.10  (0.91-10.51) | 5.06  (1.06-24.13) | 0.016 | 1.54  (0.49-4.80) | 2.75  (0.79-9.60) | 4.62  (0.97-22.01) | 0.027 |
| Combined^c^ | 1.81  (0.66-4.91) | 4.54  (1.66-12.47) | 9.85  (3.39-28.62) | <0.0001 | 1.50  (0.53-4.19) | 3.66  (1.28-10.46) | 7.57  (2.45-23.42) | 0.0002 | 1.54  (0.55-4.32) | 3.48  (1.20-10.08) | 7.96  (2.58-24.56) | 0.0002 |
|  |  |  |  |  |  |  |  |  |  |  |  |  |
| **Subcortical WMH** |  |  |  |  |  |  |  |  |  |  |  |  |
| **Recurrent stroke** |  |  |  |  |  |  |  |  |  |  |  |  |
| OXVASC | 1.34  (0.77-2.33) | 2.90  (1.67-5.01) | 2.04  (1.02-4.08) | 0.001 | 1.21  (0.68-2.13) | 2.45  (1.35-4.42) | 1.69  (0.81-3.55) | 0.017 | 1.23  (0.70-2.18) | 2.26  (1.25-4.08) | 1.69  (0.80-3.54) | 0.024 |
| HKU | 1.19  (0.50-2.82) | 2.02  (0.85-4.80) | 1.64  (0.65-4.14) | 0.046 | 1.06  (0.45-2.54) | 1.64  (0.68-3.94) | 1.23  (0.48-3.14) | 0.26 | 1.09  (0.46-2.60) | 1.74  (0.72-4.19) | 1.34  (0.52-3.44) | 0.16 |
| Combined^c^ | 1.36  (0.87-2.13) | 2.55  (1.62-3.99) | 1.95  (1.16-3.27) | 0.0001 | 1.13  (0.72-1.77) | 1.90  (1.20-3.02) | 1.40  (0.82-2.39) | 0.023 | 1.10  (0.70-1.73) | 1.83  (1.15-2.90) | 1.41  (0.82-2.41) | 0.022 |
| **Ischaemic stroke** |  |  |  |  |  |  |  |  |  |  |  |  |
| OXVASC | 1.12  (0.62-2.01) | 2.74  (1.56-4.83) | 1.92  (0.94-3.94) | 0.002 | 1.05  (0.57-1.93) | 2.47  (1.33-4.56) | 1.74  (0.80-3.76) | 0.015 | 1.06  (0.58-1.94) | 2.20  (1.20-4.07) | 1.66  (0.77-3.59) | 0.027 |
| HKU | 0.93  (0.39-2.24) | 1.71  (0.71-4.10) | 1.19  (0.45-3.12) | 0.15 | 0.82  (0.34-2.00) | 1.36  (0.56-3.31) | 0.87  (0.32-2.31) | 0.54 | 0.86  (0.35-2.09) | 1.49  (0.61-3.64) | 1.00  (0.38-2.69) | 0.31 |
| Combined^c^ | 1.14  (0.72-1.83) | 2.36  (1.48-3.76) | 1.64  (0.95-2.85) | 0.001 | 0.95  (0.59-1.53) | 1.79  (1.11-2.88) | 1.20  (0.68-2.12) | 0.058 | 0.93  (0.58-1.50) | 1.69  (1.05-2.73) | 1.21  (0.68-2.14) | 0.058 |
| **Intracerebral haemorrhage** |  |  |  |  |  |  |  |  |  |  |  |  |
| OXVASC | 6.48  (0.75-55.61) | 5.33  (0.48-58.87) | 4.68  (0.29-75.67) | 0.21 | 3.99  (0.46-34.70) | 2.57  (0.22-29.72) | 1.78  (0.10-30.91) | 0.82 | 4.72  (0.51-43.82) | 2.58  (0.20-34.12) | 2.23  (0.12-42.74) | 0.79 |
| HKU^e^ | - | - | - |  | - | - | - |  | - | - | - |  |
| Combined^c^ | 7.22  (0.92-59.97) | 7.54  (0.91-62.71) | 9.89  (1.14-85.57) | 0.052 | 5.75  (0.72-45.68) | 5.38  (0.64-45.37) | 6.64  (0.75-58.89) | 0.20 | 5.66  (0.71-44.94) | 5.37  (0.64-45.20) | 6.69  (0.75-59.61) | 0.19 |

^a^Compared with no WMH as reference ^b^Hypertension, hyperlipidaemia, diabetes mellitus, atrial fibrillation smoking history ^c^Also adjusted for Centre

^d^No intracerebral haemorrhages occurred in OXVASC patients with periventricular WMH grade 0

^e^No intracerebral haemorrhages occurred in HKU patients with subcortical WMH grade 0

Abbreviations: HR=hazard ratio, CI=confidence interval, WMH=white matter hyperintensity

**Table e-9. Cox regression analyses of risk of recurrent stroke for different burden of perivascular spaces vs. <11 perivascular spaces**

|  | **Unadjusted HR (95% CI)^a^** | | | **HR (95% CI) adjusted for age and sex^a^** | | | **HR (95% CI) adjusted for age, sex and vascular risk factors^a,b^** | | |
| --- | --- | --- | --- | --- | --- | --- | --- | --- | --- |
| **Basal ganglia PVSs** | **11-20** | **>20** | **p_trend_** | **11-20** | **>20** | **p_trend_** | **11-20** | **>20** | **p_trend_** |
| **Recurrent stroke** |  |  |  |  |  |  |  |  |  |
| OXVASC | 1.36 (0.79-2.32) | 2.45 (1.52-3.95) | 0.0003 | 1.16 (0.65-2.05) | 2.03 (1.19-3.46) | 0.01 | 1.10 (0.62-1.95) | 1.89 (1.10-3.23) | 0.023 |
| HKU | 1.46 (0.95-2.24) | 2.95 (1.70-5.11) | 0.0002 | 1.11 (0.72-1.73) | 1.97 (1.11-3.49) | 0.05 | 1.20 (0.77-1.87) | 2.32 (1.29-4.15) | 0.013 |
| Combined^c^ | 1.40 (1.00-1.95) | 2.62 (1.83-3.77) | <0.0001 | 1.11 (0.78-1.57) | 1.92 (1.30-2.83) | 0.003 | 1.13 (0.79-1.60) | 1.94 (1.30-2.88) | 0.003 |
| **Ischaemic stroke** |  |  |  |  |  |  |  |  |  |
| OXVASC | 1.24 (0.70-2.20) | 2.36 (1.43-3.89) | 0.001 | 1.11 (0.60-2.04) | 2.08 (1.18-3.65) | 0.012 | 1.04 (0.57-1.92) | 1.98 (1.13-3.49) | 0.02 |
| HKU | 1.56 (0.98-2.47) | 2.38 (1.24-4.58) | 0.004 | 1.15 (0.72-1.86) | 1.55 (0.79-3.04) | 0.22 | 1.30 (0.80-2.11) | 1.94 (0.98-3.85) | 0.056 |
| Combined^c^ | 1.40 (0.98-2.01) | 2.39 (1.61-3.54) | <0.0001 | 1.12 (0.77-1.63) | 1.77 (1.16-2.70) | 0.014 | 1.14 (0.78-1.67) | 1.81 (1.18-2.79) | 0.012 |
| **Intracerebral haemorrhage** |  |  |  |  |  |  |  |  |  |
| OXVASC | 2.55 (0.51-12.69) | 3.31 (0.66-16.59) | 0.13 | 1.39 (0.25-7.75) | 1.63 (0.29-9.35) | 0.58 | 1.05 (0.18-6.17) | 0.98 (0.15-6.31) | 0.98 |
| HKU | 1.01 (0.32-3.17) | 5.00 (1.73-14.43) | 0.019 | 0.87 (0.27-2.83) | 4.07 (1.30-12.76) | 0.062 | 0.79 (0.24-2.58) | 3.84 (1.20-12.29) | 0.089 |
| Combined^c^ | 1.33 (0.54-3.29) | 4.02 (1.63-9.94) | 0.007 | 1.02 (0.40-2.63) | 2.77 (1.05-7.35) | 0.074 | 0.95 (0.37-2.45) | 2.56 (0.96-6.84) | 0.11 |
|  |  |  |  |  |  |  |  |  |  |
| **Centrum semiovale PVSs** |  |  |  |  |  |  |  |  |  |
| **Recurrent stroke** |  |  |  |  |  |  |  |  |  |
| OXVASC | 1.07 (0.58-1.98) | 1.84 (1.06-3.19) | 0.016 | 0.90 (0.48-1.70) | 1.43 (0.79-2.59) | 0.13 | 0.83 (0.44-1.56) | 1.29 (0.71-2.35) | 0.22 |
| HKU | 1.02 (0.69-1.50) | 0.39 (0.15-0.97) | 0.15 | 1.02 (0.70-1.50) | 0.37 (0.15-0.92) | 0.12 | 1.07 (0.73-1.58) | 0.37 (0.15-0.94) | 0.16 |
| Combined^c^ | 1.02 (0.73-1.41) | 1.15 (0.78-1.68) | 0.52 | 0.92 (0.66-1.28) | 0.91 (0.61-1.35) | 0.60 | 0.92 (0.66-1.28) | 0.89 (0.59-1.32) | 0.54 |
| **Ischaemic stroke** |  |  |  |  |  |  |  |  |  |
| OXVASC | 1.20 (0.63-2.26) | 1.72 (0.95-3.10) | 0.053 | 1.04 (0.54-2.01) | 1.41 (0.75-2.67) | 0.21 | 0.94 (0.49-1.82) | 1.27 (0.68-2.40) | 0.33 |
| HKU | 1.04 (0.68-1.59) | 0.29 (0.09-0.93) | 0.14 | 1.04 (0.68-1.59) | 0.27 (0.08-0.87) | 0.11 | 1.12 (0.73-1.72) | 0.28 (0.09-0.92) | 0.18 |
| Combined^c^ | 1.07 (0.75-1.52) | 1.07 (0.70-1.63) | 0.73 | 0.96 (0.67-1.37) | 0.85 (0.55-1.31) | 0.46 | 0.96 (0.67-1.37) | 0.83 (0.54-1.28) | 0.42 |
| **Intracerebral haemorrhage** |  |  |  |  |  |  |  |  |  |
| OXVASC | - | 2.89 (0.59-14.17) | 0.087 | - | 1.37 (0.25-7.46) | 0.28 | - | 1.37 (0.21-8.93) | 0.32 |
| HKU | 0.90 (0.36-2.27) | 0.83 (0.18-3.83) | 0.77 | 0.90 (0.36-2.26) | 0.80 (0.17-3.71) | 0.75 | 0.86 (0.34-2.17) | 0.75 (0.16-3.51) | 0.67 |
| Combined^c^ | 0.71 (0.30-1.72) | 1.65 (0.65-4.20) | 0.43 | 0.67 (0.28-1.61) | 1.35 (0.51-3.57) | 0.70 | 0.68 (0.28-1.66) | 1.34 (0.50-3.55) | 0.71 |

^a^Compared with <11 PVSs as reference

^b^Hypertension, hyperlipidaemia, diabetes mellitus, atrial fibrillation smoking history

^c^Also adjusted for Centre

Abbreviations: HR=hazard ratio, CI=confidence interval, PVS=perivascular space

Table e-10. Prognostic value of the Total Small Vessel Disease Score^a^ in prediction of recurrent strokes in OXVASC and HKU (external validation)

| **Total Small Vessel Disease Score** | **Events / patients** | **Unadjusted HR (95% CI)** | **p_trend_** | **Age and sex adjusted HR (95% CI)** | **p_trend_** | **Multi-variate^b^ adjusted HR (95% CI)** | **p_trend_** | **c-statistic**  **(95% CI)** | **p** |
| --- | --- | --- | --- | --- | --- | --- | --- | --- | --- |
| **OXVASC** |  |  |  |  |  |  |  |  |  |
| 0 | 27/387 | 1 | <0.0001 | 1 | 0.0002 | 1 | 0.001 | 0.60 (0.54-0.67) | 0.001 |
| 1 | 16/293 | 0.99 (0.53-1.84) |  | 0.87 (0.45-1.67) |  | 0.83 (0.43-1.60) |  |  |  |
| 2 | 24/215 | 1.98 (1.14-3.46) |  | 1.67 (0.90-3.10) |  | 1.51 (0.81-2.81) |  |  |  |
| 3 | 16/103 | 2.94 (1.58-5.48) |  | 2.44 (1.22-4.87) |  | 2.18 (1.10-4.34) |  |  |  |
| 4 | 7/30 | 4.45 (1.93-10.27) |  | 3.73 (1.54-9.03) |  | 3.38 (1.39-8.21) |  |  |  |
| **HKU** |  |  |  |  |  |  |  |  |  |
| 0 | 13/172 | 1 | <0.0001 | 1 | 0.002 | 1 | 0.001 | 0.61 (0.56-0.67) | 0.0001 |
| 1 | 22/286 | 1.05 (0.53-2.08) |  | 1.00 (0.50-1.98) |  | 1.06 (0.53-2.13) |  |  |  |
| 2 | 29/263 | 1.59 (0.83-3.06) |  | 1.29 (0.67-2.49) |  | 1.38 (0.71-2.69) |  |  |  |
| 3 | 31/199 | 2.25 (1.18-4.30) |  | 1.73 (0.90-3.33) |  | 1.96 (1.01-3.82) |  |  |  |
| 4 | 14/54 | 4.01 (1.89-8.54) |  | 2.78 (1.29-6.01) |  | 3.15 (1.44-6.86) |  |  |  |

^a^1 point allocated for: presence of lacunes, microbleeds, moderate-severe (>10) PVSs in basal ganglia, periventricular WMH Fazekas 3 and/or deep WMH Fazekas 2-3

^b^Adjusted for age, sex, hypertension, hyperlipidaemia, diabetes, atrial fibrillation and smoking

Abbreviations: HR=hazards ratio, CI=confidence interval; PVS=perivascular space; WMH=white matter hyperintensity

Table e-11. Prognostic value of the Total Small Vessel Disease Score^a^ and Modified Total Small Vessel Disease Score^b^ in prediction of non-disabling and disabling or fatal recurrent strokes

|  | **Total Small Vessel Disease Score** | **p** | **Modified Total Small Vessel Disease Score** | **p** | **p_het_** |
| --- | --- | --- | --- | --- | --- |
| **Nondisabling recurrent ischaemic stroke** |  |  |  |  |  |
| Adjusted^c^ HR per unit increase in score (95% CI) | 1.28 (1.07-1.54) | 0.008 | 1.23 (1.07-1.42) | 0.005 | 0.73 |
| c-statistic (95% CI) | 0.56 (0.49-0.62) | 0.066 | 0.56 (0.50-0.62) | 0.056 | 1.00 |
| **Disabling / fatal recurrent ischaemic stroke** |  |  |  |  |  |
| Adjusted^c^ HR per unit increase in score (95% CI) | 1.39 (1.14-1.69) | 0.001 | 1.20 (1.03-1.39) | 0.016 | 0.24 |
| c-statistic (95% CI) | 0.65 (0.59-0.71) | <0.0001 | 0.64 (0.58-0.70) | <0.0001 | 0.82 |
|  |  |  |  |  |  |
| **Nondisabling intracerebral haemorrhage** |  |  |  |  |  |
| Adjusted^b^ HR per unit increase in score (95% CI) | 2.34 (1.15-4.74) | 0.019 | 1.87 (1.21-2.89) | 0.005 | 0.60 |
| c-statistic (95% CI) | 0.72 (0.46-0.97) | 0.068 | 0.81 (0.66-0.95) | 0.009 | 0.58 |
| **Disabling / fatal intracerebral haemorrhage** |  |  |  |  |  |
| Adjusted^c^ HR per unit increase in score (95% CI) | 1.42 (0.99-2.05) | 0.060 | 1.61 (1.27-2.04) | <0.0001 | 0.57 |
| c-statistic (95% CI) | 0.63 (0.51-0.75) | 0.032 | 0.68 (0.55-0.81) | 0.003 | 0.58 |

^a^1 point allocated for: presence of lacunes, microbleeds, moderate-severe (>10) PVSs in basal ganglia, periventricular WMH Fazekas 3 and/or deep WMH Fazekas 2-3

^b^1 point allocated for: presence of lacunes, 1-4 microbleeds, frequent-severe (>20) PVSs in basal ganglia, moderate WMH (total periventricular + subcortical WMH grade 3-4), 2 points given for ≥5 microbleeds and severe WMH (total periventricular + subcortical WMH grade 5-6)

^c^Adjusted for age, sex, hypertension, hyperlipidaemia, diabetes, atrial fibrillation and smoking

Abbreviations: HR=hazards ratio, CI=confidence interval; PVS=perivascular space; WMH=white matter hyperintensity

Table e-12. Prognostic value of modified Total Small Vessel Disease Score^a^ (internal validation)

| **Modified Total Small Vessel Disease Score** | **Events / patients** | **Unadjusted HR (95% CI)** | **p_trend_** | **Age and sex adjusted HR (95% CI)** | **p_trend_** | **Multi-variate^b^ adjusted HR (95% CI)** | **p_trend_** | **c-statistic**  **(95% CI)** | **p** |
| --- | --- | --- | --- | --- | --- | --- | --- | --- | --- |
| **Recurrent stroke** |  |  |  |  |  |  |  |  |  |
| 0 | 52/824 | 1 | <0.0001 | 1 | <0.0001 | 1 | <0.0001 | 0.62 (0.58-0.66) | <0.0001 |
| 1 | 54/593 | 1.60 (1.09-2.35) |  | 1.42 (0.96-2.09) |  | 1.35 (0.92-1.99) |  |  |  |
| 2 | 41/280 | 2.65 (1.76-3.99) |  | 2.09 (1.37-3.21) |  | 1.95 (1.27-3.00) |  |  |  |
| 3 | 26/170 | 2.84 (1.77-4.56) |  | 2.30 (1.42-3.73) |  | 2.30 (1.41-3.73) |  |  |  |
| 4 | 11/79 | 2.83 (1.47-5.43) |  | 2.35 (1.22-4.54) |  | 2.34 (1.21-4.53) |  |  |  |
| 5 | 9/42 | 4.13 (2.03-8.39) |  | 3.13 (1.52-6.45) |  | 3.10 (1.50-6.40) |  |  |  |
| 6 | 6/14 | 7.73 (3.32-18.01) |  | 6.01 (2.55-14.15) |  | 6.41 (2.70-15.20) |  |  |  |
|  |  |  |  |  |  |  |  |  |  |
| **Recurrent ischaemic stroke** |  |  |  |  |  |  |  |  |  |
| 0 | 46/824 | 1 | <0.0001 | 1 | 0.0003 | 1 | 0.0003 | 0.60 (0.56-0.65) | <0.0001 |
| 1 | 49/593 | 1.63 (1.09-2.44) |  | 1.44 (0.96-2.17) |  | 1.38 (0.91-2.08) |  |  |  |
| 2 | 37/280 | 2.67 (1.73-4.13) |  | 2.11 (1.35-3.32) |  | 1.97 (1.25-3.10) |  |  |  |
| 3 | 20/170 | 2.42 (1.43-4.09) |  | 1.96 (1.14-3.35) |  | 1.94 (1.13-3.32) |  |  |  |
| 4 | 10/79 | 2.88 (1.45-5.71) |  | 2.38 (1.19-4.76) |  | 2.40 (1.20-4.80) |  |  |  |
| 5 | 5/42 | 2.48 (0.98-6.24) |  | 1.90 (0.74-4.83) |  | 1.92 (0.75-4.92) |  |  |  |
| 6 | 3/14 | 4.23 (1.32-13.62) |  | 3.27 (1.01-10.61) |  | 3.62 (1.11-11.84) |  |  |  |
|  |  |  |  |  |  |  |  |  |  |
| **Intracerebral haemorrhage** |  |  |  |  |  |  |  |  |  |
| 0 | 6/824 | 1 | <0.0001 | 1 | <0.0001 | 1 | <0.0001 | 0.71 (0.60-0.81) | 0.0001 |
| 1 | 5/593 | 1.28 (0.39-4.20) |  | 1.13 (0.34-3.77) |  | 1.05 (0.32-3.51) |  |  |  |
| 2 | 4/280 | 2.23 (0.63-7.94) |  | 1.79 (0.48-6.65) |  | 1.70 (0.46-6.35) |  |  |  |
| 3 | 6/170 | 5.59 (1.80-17.35) |  | 4.55 (1.40-14.71) |  | 4.64 (1.43-15.07) |  |  |  |
| 4 | 1/79 | 2.21 (0.27-18.42) |  | 1.91 (0.23-16.08) |  | 1.86 (0.22-15.71) |  |  |  |
| 5 | 4/42 | 15.87 (4.45-56.55) |  | 12.10 (3.19-45.97) |  | 9.91 (2.59-37.93) |  |  |  |
| 6 | 3/14 | 27.55 (6.87-110.50) |  | 21.57 (5.12-90.80) |  | 21.48 (4.96-93.02) |  |  |  |

^a^1 point allocated for: presence of lacunes, 1-4 microbleeds, frequent-severe (>20) perivascular spaces in basal ganglia, moderate WMH (total periventricular + subcortical WMH grade 3-4), 2 points given for ≥5 microbleeds and severe WMH (total periventricular + subcortical WMH grade 5-6)

^b^Adjusted for age, sex, hypertension, hyperlipidaemia, diabetes, atrial fibrillation and smoking

Abbreviations: HR=hazards ratio, CI=confidence interval
